# Design Guidelines for Sensors Based on Spiral Resonators

**DOI:** 10.3390/s22052071

**Published:** 2022-03-07

**Authors:** Mahmoud Elgeziry, Filippo Costa, Simone Genovesi

**Affiliations:** Dipartimento di Ingegneria dell’Informazione, Università di Pisa, 56126 Pisa, Italy; mahmoud.elgeziry@phd.unipi.it (M.E.); filippo.costa@unipi.it (F.C.)

**Keywords:** spiral resonators, microwave sensors, metamaterials, displacement sensors, distance sensors

## Abstract

Wireless microwave sensors provide a practical alternative where traditional contact-based measurement techniques are not possible to implement or suffer from performance deterioration. Resonating elements are commonly used in these sensors as the sensing concept relies on the resonance properties of the employed structure. This work presents some simple guidelines for designing displacement sensors based on spiral resonator (SR) tags. The working principle of this sensor is based on the variation of the coupling strength between the SR tag and a probing microstrip loop with the distance between them. The performance of the sensor depends on the main design parameters, such as tag dimensions, filling factor, number of turns, and the size of probing loop. The guidelines provided herein can be used for the initial phase of the design process by helping to select a preliminary set of parameters according to the desired application requirements. The provided conclusions are supported using electromagnetic simulations and analytical expressions. Finally, a corrected equivalent circuit model that takes into account the phenomenon of the resonant frequency shift at small distances is provided. The findings are compared against experimental measurements to verify their validity.

## 1. Introduction

In recent years, the use of metamaterials in sensing applications is becoming more popular, as evidenced by the increasing number of published works employing these engineered structures in sensor design [1,2,3]. Contrarily to classical sensors, which rely on physical contact between the reading instrument and the sensing structure, these sensors can be interrogated wirelessly. Moreover, these new sensors may help to improve the performance by adding more degrees of freedom, which leads to novel and practical advantages, such as higher sensitivity or operation in harsh environments. The operating principle of these sensors based on resonating elements relies on linking a change in the physical property to be measured (measurand) with a change in the resonant behavior of the components. This could either be a change in the resonant frequency (frequency-modulation) or a change in the amplitude at resonance (amplitude-modulation) [4]. An interesting review of the use of metamaterial unit-cell-based components in sensing provided in [1], with a special focus on the well-known split ring resonator (SRR) geometry first proposed by Pendry [5], provides a good understanding of the working principle as well as some considerations in the design of this kind of sensors. In frequency-modulated sensors the working principle relies on the simple fact that the resonant frequency is changed due to a change in the measurand; however, the measurement is carried out by means of a wide-band frequency sweep which increases the complexity and cost of the electronics required for signal generation and detection. Frequency-modulated sensors have been used in a variety of applications, such as strain sensors [6], motion detection [7], liquid detection [8], rotation sensors [9,10], methanol concentration [11], and metal detection [12]. These sensors, based on metamaterial unit cell geometries, are capable of providing real-time monitoring of various physical properties and they can operate without any wired connection between the reader and the sensor. Another commonly used geometry in wireless sensors is the spiral resonator (SR) geometry. Spiral resonators have found applications in many fields, such as wearables [13,14], biomedical [13,15], strain [14], eddy current [16], and displacement [17,18,19] sensing.

Equivalent circuit models for the microwave sensor geometries have been presented in numerous works in the literature regarding the evaluation of the equivalent lumped circuit parameters [20,21,22,23,24,25,26]. However, the focus of these works is often the unique implementation itself, that is designed and developed for a specific application with a strict set of limitations and constraints regarding the operating frequency or sensor reading range. This is a drawback and hinders the possibility for reproducing or readapting the implementation for a different set of operating conditions or application requirements. When approaching the design problem, the designer must make trade-offs and compromises according to the requirements and constraints of the application in hand. In SR-based distance sensors, examples of such design targets could be related to the maximum sensor range, the operating frequency, or the robustness to lateral displacements. Therefore, the main aim of this article is to provide the designer with sufficient understanding of the sensor working principle and the effects of varying the design parameters and how to optimize them for the required application. The distance sensor proposed in this paper is based on an SR tag etched on a dielectric substrate. The tag is interrogated by a microstrip probing loop that acts as the antenna of the reader. The sensor is capable of providing real-time estimates of the distance between the probe and the tag by means of a measurement of the real part of the input impedance at the loop terminals. The approach adopted in this work is to investigate the effect of the proposed sensor’s parameters using numerical simulations that are supported by approximated analytical expressions. The sensor design and the theory of the working principle are introduced in Section 2 along with a model for the sensor using equivalent circuits. Section 3 treats the relation between the sensor performance and the geometric parameters of the SR and the probing loop. A corrected equivalent circuit model is proposed in Section 4 that accurately models the phenomenon of the resonant frequency shift at small distances. This circuit model is verified using experimental measurements in Section 5. Finally, the concluding remarks are discussed in Section 6.

## 2. Sensor Design and Theory

**Figure 1:**
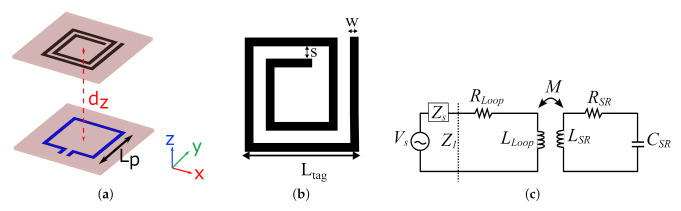
SR-based distance sensor presented in [27]. (**a**) 3D view of the sensor setup. (**b**) Top view of the SR. (**c**) Equivalent circuit model of the sensor; the SR is modeled using the series RLC circuit (right side) coupled to the probing loop by the mutual inductance, *M*.

The working principle of the proposed distance sensor based on a planar SR relies on the near-field inductive coupling between the SR and the microstrip probing loop that acts as the antenna of the reader. The strength of this coupling was shown to be inversely proportional to the distance between them. This concept was exploited to retrieve an accurate estimate of the distance by simply measuring the input impedance, whose value depends on the strength of the mutual coupling between the two components, at the probe terminals. The SR-based sensor is shown in Figure 1a where the SR and the probing loop are separated by a normal distance $d_{z}$. Figure 1b shows a generic 2-turn square-shaped SR having side length $L_{tag}$, trace width w, and trace separation s. The sensor can be modeled using the equivalent circuit shown in Figure 1c where the SR is represented by a series RLC circuit that is coupled to the probing loop through the mutual inductance term, *M* [27].

The aforementioned coupling between the probe and the SR occurs at the resonant frequency of the SR, which can be expressed in terms of the equivalent circuit lumped parameters shown in Figure 1c: inductance, $L_{SR}$, and capacitance, $C_{SR}$, as in (1), which in turn depend on the geometrical parameters ($L_{tag}$, w, and s, *N*) for the case of the SR shown in Figure 1b.

(1) $$f_{0}=\frac{1}{2\pi \sqrt{L_{SR}C_{SR}}}.$$

**Figure 2:**
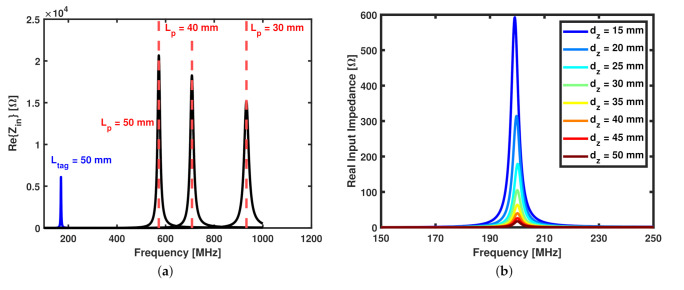
(**a**) Real input impedance vs. frequency for the standalone square probing loop with side length 30 mm, 40 mm, and 50 mm (black lines), and the case with an SR in close proximity having $L_{tag}$ = 50 mm shown for $d_{z}=$ 1 mm. (**b**) Real input impedance vs. frequency for sensor with w=s= 2 mm, N= 2 turns, and $L_{p}=L_{tag}=$ 50 mm plotted at various values of $d_{z}$, showing the dependence on the normal distance.

One important consideration in the design of the SR already outlined in is that the SR parameters must be chosen so that the resonant frequency is far from the self-resonant frequency of the probe. This consideration can be shown by analyzing the results from the electromagnetic (EM) model of the system created in CST Microwave Studio. A summary of the results of this preliminary simulation is shown in Figure 2a, where the spectrum of the real value of the input impedance of the standalone probing loop with different side lengths, $L_{p}$, is plotted in black solid lines and the resonant frequency is marked by the vertical red dashed lines for each value of $L_{p}$. The blue line plotted in the same figure shows the resonance due to the presence of the square-shaped SR with side length $L_{tag}\;=$ 50 mm and $L_{p}=$ 30, 40, and 50 mm. Figure 2a shows that the resonance due to the spiral resonator occurs at a frequency that is significantly lower than that due to the self-resonance of the probing loop. It is worth nothing that the blue line is plotted for $d_{z}=$ 1 mm and it follows that the amplitude at resonance depends on the value of $d_{z}$, as shown in the plot in Figure 2b, where the amplitude at resonance for a square SR coupled with a square probing loop both having side length equal to 50 mm decreases as the normal distance, $d_{z}$, between them increases.

## 3. Maximizing the Sensing Range

The sensing range can be defined as the maximum distance (between the SR and the loop) at which there is an appreciable change in the measured real input impedance at the probe terminals for a change in this distance. The aim of this section is to provide useful guidelines for designing SR-based distance sensors optimized for maximum normal distance sensing range. The input impedance can be calculated using the expression in (2) in terms of the lumped equivalent circuit parameters [22]:

(2) $$Z_{1}=R_{Loop}+j\omega L_{Loop}+\frac{\omega^{2}M^{2}}{j\omega L_{SR}+\frac{1}{j\omega C_{SR}}+R_{SR}}.$$

where ω is the operating frequency in radians per second, and $L_{Loop}$ and $R_{Loop}$ are the lumped inductance and resistance, respectively, of the probing loop. The lumped parameters $L_{SR}$, $C_{SR}$, and $R_{SR}$ model the inductance, capacitance, and the resistance of the SR, whereas *M* represents the mutual inductance between the SR and the probe. The lumped circuit parameters’ equivalent model is valid as long as the size of the components is much smaller than the wavelength at the operating frequency [21]. By looking at the expression in (2), it is straightforward to observe how a change in the mutual inductance, which is inversely proportional to the normal distance ($d_{z}$) between the SR and the probe, causes a change in the measured input impedance at the probing loop terminals.

At this point, the sensitivity, *S*, in (3) is defined as the change in the input impedance divided by the change in the normal distance.

(3) S=Re(Zi)−Re(Zi+1)dzi−dzi+1

where $Z_{i}$ is the input impedance at resonance measured at the loop terminals for a certain value of the tag-reader distance $d_{z_{i}}$.

### 3.1. Scaling SR and Probe Dimensions

**Figure 3:**
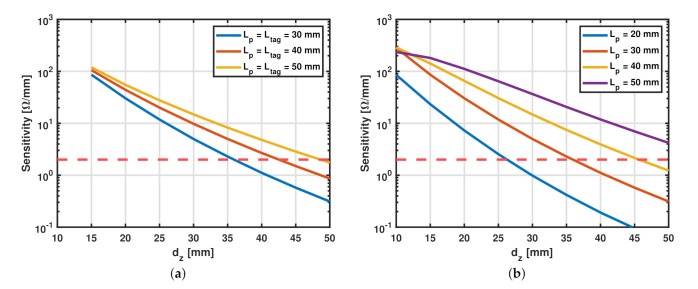
(**a**) Sensitivity vs. normal distance for different sizes of the sensor while keeping the side length of the probe and the SR equal. (**b**) Sensitivity vs. normal distance while varying the probe dimensions for a constant side length of the SR, $L_{tag}=$ 30 mm. The dashed red lines represent a threshold sensitivity of 2 Ω/mm.

A design consideration regarding the range of the sensor was presented in [27], where it was shown that increasing the size of the SR and the probing loop increases the sensing range of this device. This concept can be better explained again by considering the scenario with square-shaped SR and probing loop, both having the same side length. An EM simulation was carried out for the distance sensor of different sizes, and as observed from Figure 3a where *S* is plotted against the normal distance at different values of the side length of the SR and the probe. It can be observed that the sensitivity at the same value of $d_{z}$ is larger as the size of the sensor becomes bigger. The minimum change in impedance depends on the characteristics of the measurement instrument used. For a supposed threshold instrument measurement sensitivity of 2 Ω/mm, shown by the dashed red line in Figure 3a, one can observe that the maximum sensing range of the sensor is more or less comparable to the side length of the SR.

Even though it is evident that scaling up the size of both the SR and the probe increases the sensitivity at large values of $d_{z}$, and consequently increases the range of the sensor, further analyses have shown that this is not the optimal method for increasing the range. This is explained in detail using data obtained from numerical simulations in Section 3.2 and Section 3.3.

### 3.2. Optimizing Probe Dimensions

In some applications the available space for the SR tag might be limited and imposed as a constraint. This case with a fixed tag size is analyzed thoroughly in this subsection with the aim of providing the reader with an understanding of the process and consequently guidance for optimizing the probe design. Firstly, a parametric simulation was performed for a square-shaped probing loop ($L_{p}=$ 30 mm) and an SR with side length $L_{tag}=$ 30 mm while varying the value $d_{z}$ from 1 mm to 50 mm. The substrates for both the tag and the SR are also square-shaped with a side length of 10 cm and are made of FR-4. The same simulation was performed for different values of $L_{p}$ to study the effect of the relative size between the SR and the probing loop on the strength of the coupling at small and large values of $d_{z}$. The sensitivity of the sensor is plotted versus the normal distance in Figure 3b and it can be observed that at large values of $d_{z}$, for a constant $L_{tag}$, increasing the size of the probing loop increases the sensitivity. This is advantageous for applications where maximizing the sensing range is of interest.

The increase in the input impedance at large distances when increasing the size of the probing loop is owed to the increase in the strength of the mutual inductive coupling between the probe and the SR. This follows from the analytical expressions for calculating the mutual inductance between coaxial spiral coils based on the Biot–Savart law for calculating the axial magnetic field component of the coil. A pair of coaxial square single-turn filament coils separated by a normal distance $d_{z}$ are considered. For a unit current, the mutual inductance, *M*, depends on the side lengths of the square coils and the distance between them. *R* is defined as the ratio between the side lengths of the probe and the tag.

(4) $$R=\frac{L_{p}}{L_{tag}}.$$

The mutual inductance between the two coils can then be expressed in terms of the ratio *R* [26].

(5) $$\begin{array}{cc} \; & M=\frac{2\mu }{\pi }[r_{p}+r_{m}-2r_{r}+\;\\ \; & +\frac{L_{tag}\left(R+1\right)}{2}\left[\mathrm{atanh}\frac{L_{tag}\left(R+1\right)}{2r_{r}}-\mathrm{atanh}\frac{L_{tag}\left(R+1\right)}{2r_{p}}\right]+\;\\ \; & +\frac{L_{tag}\left(R-1\right)}{2}\left[\mathrm{atanh}\frac{L_{tag}\left(R-1\right)}{2r_{r}}-\mathrm{atanh}\frac{L_{tag}\left(R-1\right)}{2r_{p}}\right]]. \end{array}$$

where

(6) $$r_{p}=\sqrt{\frac{L_{tag}^{2}}{2}{\left(R+1\right)}^{2}+d_{z}^{2}}$$

(7) $$r_{m}=\sqrt{\frac{L_{tag}^{2}}{2}{\left(R-1\right)}^{2}+d_{z}^{2}}$$

(8) $$r_{r}=\sqrt{\frac{L_{tag}^{2}}{2}\left(R^{2}+1\right)+d_{z}^{2}}$$

**Figure 4:**
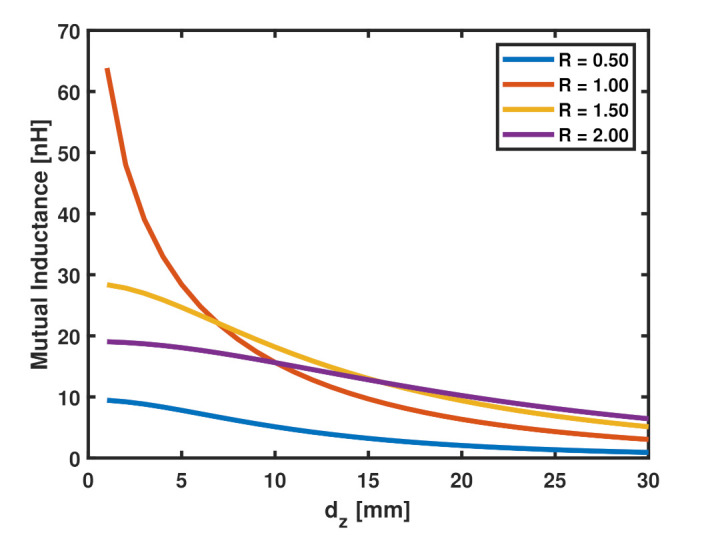
Mutual inductance between two coaxial single-turn square-shaped filament coils against the normal distance between them, $d_{z}$, for values of *r* for a constant value of $L_{tag}=$ 30 mm, w=s= 2 mm.

For the same tag size (constant $L_{tag}$), increasing the size of the loop ($L_{p}$) increases the mutual inductance at large values of $d_{z}$. To demonstrate this concept, the mutual inductance, *M*, between two coaxial single-turn square coils calculated from (5) is plotted against the normal distance for different values of *R* in Figure 4 for $L_{tag}=$ 30 mm. As evident from the figure, at small $d_{z}$ the coupling is strongest for $L_{p}=L_{tag}$ (R= 1), while for large values of $d_{z}$ increasing the size of the probe increases the strength of the coupling which explains the trend in Figure 3b.

It is concluded that for applications where the size of the SR is a constraint, the sensing range (sensitivity at large normal distances) can be maximized by increasing the size of the probe compared to the size of the tag. The opposite is true for a scenario with restrictions on the size of the probe; in this case, the size of the SR must be decreased to increase the sensing range, whereas for applications requiring high sensitivity at small normal distances, the probing loop must be designed to have a side length close to that of the tag, which is consistent with previous works in the literature regarding the coupling coefficient optimization in wireless power transfer [28,29].

### 3.3. Robustness to Lateral Displacements

Another key aspect to consider in the design of SR-based normal distance sensors, especially in applications where the axial alignment between the SR and the probe cannot be guaranteed, is the robustness to a potential lateral misalignment. It follows from the definition of the working principle of this sensor in Section 2 that any change in the lateral displacement from the coaxial condition causes a decrease in the magnetic flux intercepted by the tag, and therefore decreases the coupling strength between the probe and the SR, which is translated into a decrease in the input impedance at the probing loop terminals.

**Figure 5:**
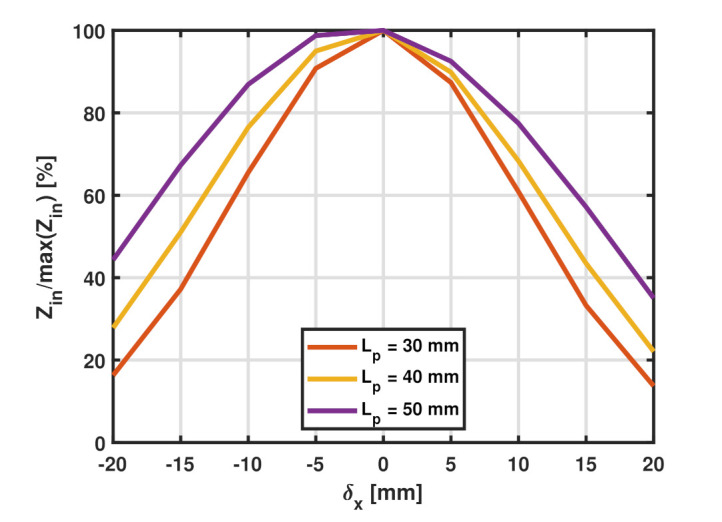
Input impedance variation, expressed as percentage of maximum real input impedance, as the degree of lateral misalignment changes for an SR with $L_{tag}=$ 30 mm interrogated by a probe of varying side length.

For a given value of normal distance, for instance $d_{z}=$ 20 mm, the maximum real input impedance is observed in the aligned case ($\delta_{x}=$ 0 mm), where $\delta_{x}$ represents the lateral distance between the axis of the probing loop and the SR. As the SR tag is laterally displaced over the probe ($\left|\delta_{x}\right|\neq$ 0 mm), the measured impedance decreases accordingly. This is shown in Figure 5, where the y-axis represents the real input impedance as a percentage of the maximum value (observed at $\delta_{x}=$ 0 mm) and the x-axis represents the lateral misalignment in the x-direction. The plot is shown for a constant value of $L_{tag}=$ 30 mm and varying the side length of the probe from 30 to 50 mm. It is apparent that increasing the size of the probe increases the coupling strength at the same degree of misalignment. In other words, probing the SR tag using a bigger loop increases its stability to misalignment in the lateral directions. This is a logical conclusion that follows from the discussion made in Section 3.2 that maximizing the coupling strength increases the range of displacements for which this coupling maintains its strength. From a qualitative point of view, the SR tag placed in the vicinity of a more uniform magnetic field is produced by a loop with a significantly bigger side length (high value of *R*). For the same normal distance separation, the SR experiences a more homogeneous magnetic field as *R* increases. As a result, the coupling strength is more resistant to lateral displacements. Another observation from Figure 5 is that for the same degree of misalignment the input impedance is higher for the negative $\delta_{x}$ than its positive counterpart, and this is due to the asymmetric shape of the SR.

In this section, only lateral displacements in the x-direction ($\delta_{x}$) are considered; however, since the probe and the SR are both square-shaped, the response to displacements in the y-direction ($\delta_{x}$) follows the same trend. This is an important consideration for practical scenarios where the sensor is susceptible to lateral misalignment in only one of the two directions. In this case, for decreasing the space occupied by the loop, it is possible to redesign the probing loop to be rectangular-shaped by increasing the length of the side along the direction of the expected misalignment.

The significance of the robustness to misalignment is entirely dependent on the nature of the application. In some applications it may be possible to accurately control the axial alignment, and therefore this factor is not relevant, whereas in other applications, where the axial alignment varies regularly and significantly, the design may favor the robustness to lateral displacements over other parameters such as the sensor range. One example of such a scenario is the implementation of this sensor in wearable applications, where guaranteeing the axial alignment is not possible.

### 3.4. Analysis of the Effect of Other Design Parameters

The focus of this subsection is to provide the reader with an understanding of the effect of varying the trace width (*w*), trace separation (*s*), and the number of turns (*N*) in the SR tag. It has been shown that increasing *N* leads to an increase in the inductance of the SR ($L_{SR}$) [25], and therefore its resonant frequency decreases according to (1). For this study, it is assumed that increasing the value of *N* by 1 means adding another turn inside the SR (i.e., the external perimeter of the SR remains unchanged). As a result of this decrease in the resonant frequency, the input impedance, $Z_{1}$, at the probe terminals decreases according to (2). On the other hand, adding more turns inside the SR increases the mutual inductance, *M*, between the SR and the probe, which in turn increases the value of $Z_{1}$.

**Figure 6:**
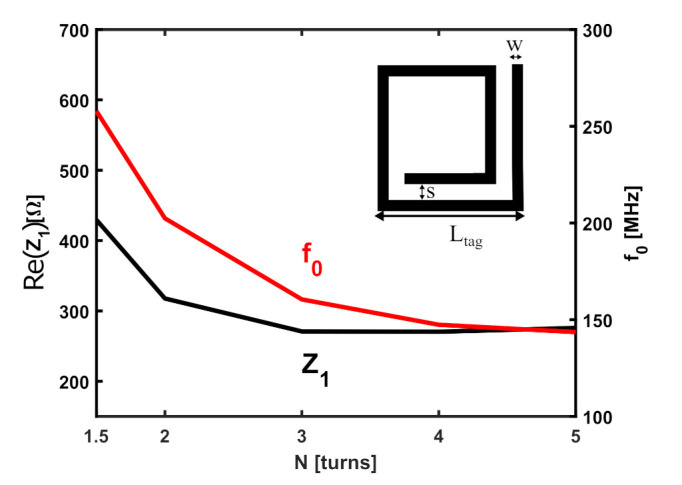
Effect of the number of turns *N* on the input impedance at the probe terminals and the resonant frequency of the SR. Results are plotted for $L_{p}=L_{tag}=$ 50 mm, w=s= 2 mm, and $d_{z}=$ 20 mm.

The results obtained from the EM simulations for varying the number of turns of the SR from 1.5 turns (shown in the top right corner of the plot) to 5 turns are illustrated in Figure 6. The simulations were carried out for the same square-shaped SR and probing loop with side lengths ($L_{p}=L_{tag}=$ 50 mm). For the purpose of simplicity, the plot in Figure 6 is shown for a fixed normal distance $d_{z}=$ 20 mm; however, the behavior is the same at other values of $d_{z}$, just shifted upwards or downwards depending on the value. It can be observed from the figure that the resonant frequency, $f_{0}$ (right y-axis—plotted with a red solid line), decreases as the number of turns increase. However, each additional turn causes a decrease in the resonant frequency smaller than the previous turn (the length of the added turn decreases as *N* increases) until it reaches a saturation point where adding a new turn does not cause a significant change in the resonant frequency. The real input impedance decreases as well the resonant frequency. Therefore, increasing the number of turns for the sensor with the presented parameters does not improve its sensitivity or range.

**Figure 7:**
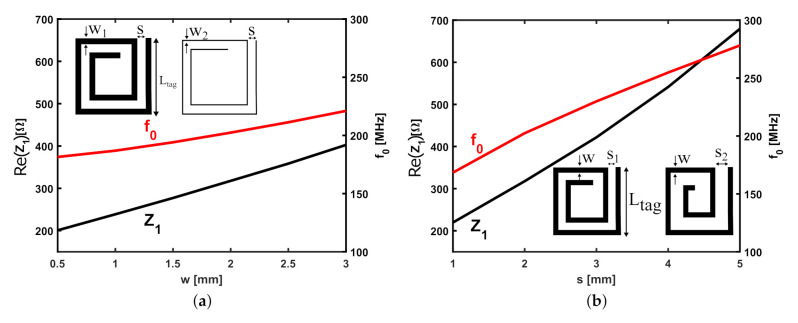
(**a**) Effect of the trace width *w* on the input impedance at the probe terminals and the resonant frequency of the SR. Results are plotted for $L_{p}=L_{tag}=$ 50 mm, s= 2 mm, N= 2 turns, and $d_{z}=$ 20 mm. (**b**) Effect of the trace separation *s* on the input impedance at the probe terminals and the resonant frequency of the SR. Results are plotted for $L_{p}=L_{tag}=$ 50 mm, w= 2 mm, N= 2 turns, and $d_{z}=$ 20 mm.

The effect of varying the trace width (*w*) and separation (*s*) is studied while keeping all the other parameters constant ($L_{p}=L_{tag}=$ 50 mm and N= 2 turns). The results are shown in Figure 7a,b, where it is evident that increasing those parameters increases the impedance at the probing loop terminals (plotted in black solid lines—left y-axis) as a result of a growth of the resonant frequency which is also plotted on the same figures (red solid lines—right y-axis). The increase in the resonant frequency can be explained by a decrease in the overall inductance of the SR for larger strip width and/or separation while keeping the external side length and the number of turns constant. The decrease in the inductance is due to a larger filling factor (i.e., the spiral resonator becomes less hollow [25,30]). Moreover, increasing the trace spacing, *s*, decreases the turn capacitance of the SR and therefore it adds another contribution to the increase in the resonant frequency. These parameters can be optimized to fine-tune the sensor for the specific application operating in the desired frequency range.

## 4. Coupling between the SR and the Loop at Short Separation Distances

**Figure 8:**
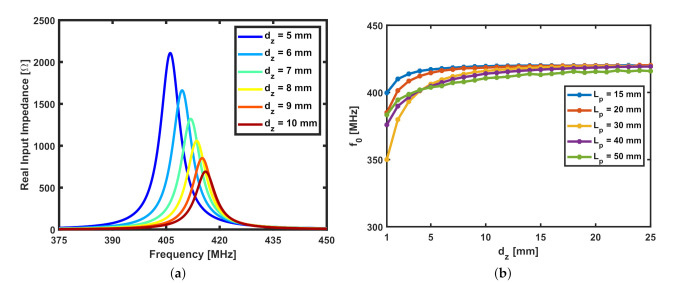
(**a**) Real input impedance vs. frequency as $d_{z}$ varies from 5 to 10 mm for the sensor with $L_{p}=L_{tag}=$ 30 mm, w=s= 2 mm, N= 2 turns. (**b**) Resonant frequency vs. $d_{z}$ for varying $L_{p}$ at $L_{tag}=$ 30 mm, w=s= 2 mm, N= 2 turns.

The equivalent circuit model presented in Figure 1c has limitations in describing the physical coupling between the spiral resonator and the probing loop at short distances. Indeed, according to the circuit model, the input impedance of the loop should be modulated by the increase of the separation distance, but numerical simulations reveal a drastic frequency shift when the spiral resonator is very close to the loop. An EM simulation was carried out for a sensor with the following design parameters: $L_{p}=L_{tag}=$ 30 mm, w=s= 2 mm, and N= 2 turns. The SR and the probing loop are modeled as perfect electric conductor (PEC) strips etched on FR-4 substrates of the same dimensions, as reported in Section 2. The real part of the input impedance was analyzed in this case starting from small values of normal distance $d_{z}$, and the results from the simulation are plotted in Figure 8a for $d_{z}$ from 5 to 10 mm. A significant decrease in the resonant frequency of the SR can be observed when the tag is in close proximity to the probe. The relation between the resonant frequency and $d_{z}$ is shown by the plot in Figure 8b for different values of $L_{p}$ at a constant SR size ($L_{tag}=$ 30 mm). The shift in the resonant frequency could also be observed in Figure 2b, as there is a slight increase in the resonant frequency of the SR as $d_{z}$ increases from 15 to 20 mm. However, as $d_{z}$ increases, the change in the frequency at which the SR resonates becomes less significant. This phenomenon can be attributed to an additional capacitance formed between the loop and the spiral resonator at short separation distances. The presence of this additional capacitance may be numerically evaluated by a thorough analysis of numerical simulations. This model may help in compensating this undesired effect especially if a single-frequency reader is employed. Clearly, the use of readers operating at a single frequency is preferable since it requires significantly simpler electronics compared to that of a reader that performs a frequency sweep.

The phenomenon of the frequency shift at small distances was reported in a few works in the literature; for example, the results published in [19] show that decreasing the normal distance causes a decrease in the first resonant frequency, whereas in [31], the range of the sensor is smaller when using a single-frequency reader due to the shift in the resonant frequency with the displacement. On the other hand, the rotation sensor in [9] suffered for perturbations in the normal distance due to the shift in the resonant frequency. Despite the frequent occurrence of this phenomenon, to the best of the authors’ knowledge, it has not been treated in the literature from a modeling point of view. Therefore, the resonant frequency dependence on the normal distance at close proximity is investigated in this section with the aim of providing the reader with an understanding of the process, and finally the equivalent circuit model previously presented in Section 3 is improved to account for this resonant frequency shift at small $d_{z}$.

### 4.1. Near-Field Capacitive Coupling

The resonant frequency of the SR depends on its equivalent capacitance and inductance as demonstrated previously in (1). Therefore, any change in these equivalent parameters causes a change in the resonant frequency. The distance-dependent capacitive coupling was addressed in [32] for the design of integrated circuits where the capacitance between a conductor strip and a ground plane varies with the distance between them. Moreover, in a more recent work [33], the capacitance between two pads in high-density communication was modeled as a function of the distance between them. The expressions developed in these two models include a term that represents the parallel plate capacitance effect as in (9) for two parallel conducting plates with surface area *A* and separated by a normal distance *d*

(9) $$C_{ppc}=\varepsilon_{0}\frac{A}{d}$$

where $\varepsilon_{0}$ is the permittivity of free space.

**Figure 9:**
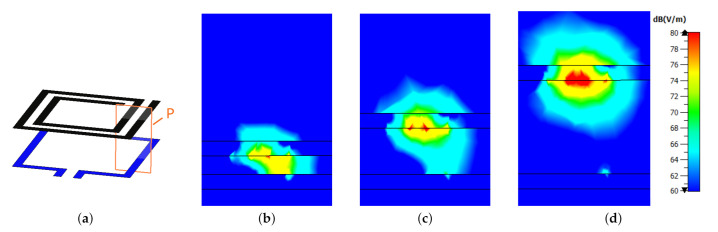
(**a**) 3D view of the sensor (dielectric substrates are hidden for better visibility) showing the section plane *P* where the electric field distribution is plotted for (**b**) $d_{z}=$ 2 mm (at 379 MHz), (**c**) $d_{z}=$ 5 mm (at 406 MHz), (**d**) $d_{z}=$ 10 mm (at 416 MHz).

Firstly, the EM simulation tool was used to plot the electric field distribution for the case where $L_{p}=L_{tag}=$ 30 mm at various values of $d_{z}$. The plots in Figure 9b–d show the average electric field distribution over a section plane *P* (shown by the orange rectangle in Figure 9a where the FR-4 substrates are hidden for better visibility). It is evident from the figures that the strength of the electric field between the loop and the SR is strongest at $d_{z}=$ 2 mm, and becomes weaker as the SR and the loop grow farther apart. It is worth noting that the electric field in each level of $d_{z}$ is plotted at the corresponding resonant frequency (379 MHz, 406 MHz, and 416 MHz, respectively). This suggests that at small distances, there is a capacitive coupling between the SR and the probe that has not been accounted for in the equivalent circuit model shown in Figure 1c. In particular, there is a missing capacitance that should be added to the model whose value is not fixed (for a set of geometric parameters) but dependent on the normal distance $d_{z}$, analogous to the parallel plate capacitor model in (9). This model is further supported by the observation that the maximum shift in the resonant frequency of the SR occurs when it has the same size as the probing loop (yellow curve in Figure 8b), i.e., maximum conductor strip overlap area.

### 4.2. Modeling of the Additional Capacitance

The capacitive coupling between the SR and the probing loop at small distances can be accounted for by adding a distance-dependent term to the expression of the capacitance of the SR as in (10), where $C_{f}$ is the native capacitance of the SR (estimated at a $d_{z}$ beyond which there is no change in the resonant frequency) according to (11) where the native SR resonant frequency, $f_{\infty }$, is obtained from numerical simulations at large $d_{z}=$ 30 mm, where this capacitive coupling effect is insignificant. The inductance is calculated in (12) according to [25] for an SR of *N* turns. The constants in (12) depend on the layout and are obtained for a square planar spiral, $\mu_{0}$ is the free space permeability, and ρ represents the filling factor and is calculated as the ratio between the difference of the external and internal diameter of the SR to their sum. The additional capacitance $C_{ppc}$ is calculated from (13) in terms of the effective dielectric permittivity of the medium $\varepsilon_{0}$ between the loop and the SR (modeled here as vacuum which is a valid first approximation since the space between the conductors is occupied as air), the equivalent conductor strip area $\tilde{A}$, and the normal distance $d_{z}$.

(10) $$C_{SR}\left(d_{z}\right)=C_{f}+C_{ppc}\left(d_{z}\right).$$

(11) $$C_{f}=\frac{1}{{\left(2\pi f_{\infty }\right)}^{2}L_{SR}}$$

(12) $$L_{SR}=2.34\mu_{0}\frac{N^{2}d_{avg}}{1+2.75\rho }$$

(13) $$C_{ppc}\left(d_{z}\right)=\varepsilon_{0}\frac{\tilde{A}}{d_{z}}.$$

**Figure 10:**
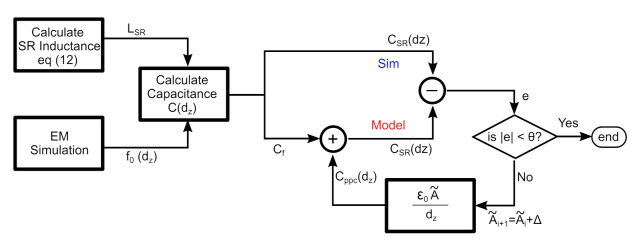
Flowchart for the proposed additional capacitance model where $f_{0}\left(d_{z}\right)$ is the resonant frequency and $C_{SR}\left(d_{z}\right)$ is the SR total capacitance, both expressed as a function of $d_{z}$, and *e* is the error between the model and simulation results. Δ is the step increment of the fitted variable $\tilde{A}$, and θ is the desired max error.

**Figure 11:**
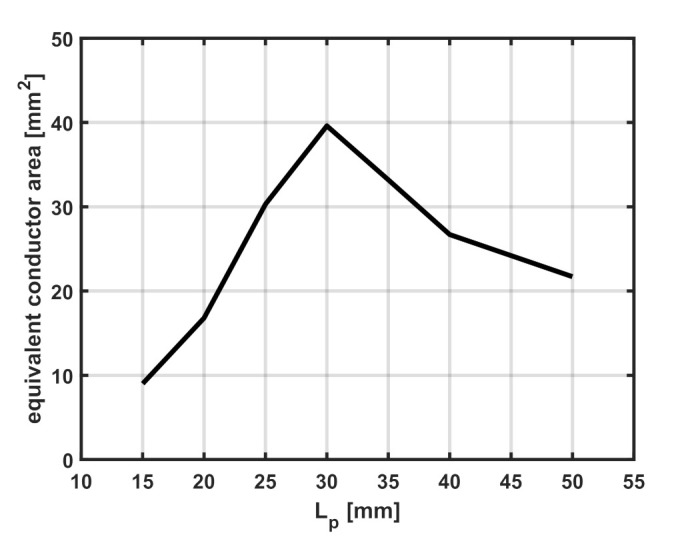
Fitted equivalent strip area ($\tilde{A}$) vs. the size of the probing loop $L_{p}$ for a constant tag size $L_{tag}=$ 30 mm.

The terms in expressions (11) and (12) depend on the geometrical parameters with the exception of $f_{0}$ that is obtained from a numerical simulation. To calculate the additional capacitance $C_{ppc}$, an algorithm based on least square fitting was developed to estimate the best fit parameters. The block diagram of the implemented algorithm is shown graphically in Figure 10, to correctly account for this distance-dependent capacitance. The fitting algorithm was run on Matlab and the results are summarized in Figure 11 and Table 1 for the values of the equivalent areas $\tilde{A}$ having the unit [m${}^{2}$] from dimensional analysis of (13).

The trend of the fitted values of the equivalent area, $\tilde{A}$, is consistent with the parallel plate capacitor model since $\tilde{A}$ reaches its maximum value when the SR and the loop have the same side length ($L_{tag}=L_{p}=$ 30 mm) where the overlap area is maximum, as shown in Figure 11, which is consistent with the physical model.

**Figure 12:**
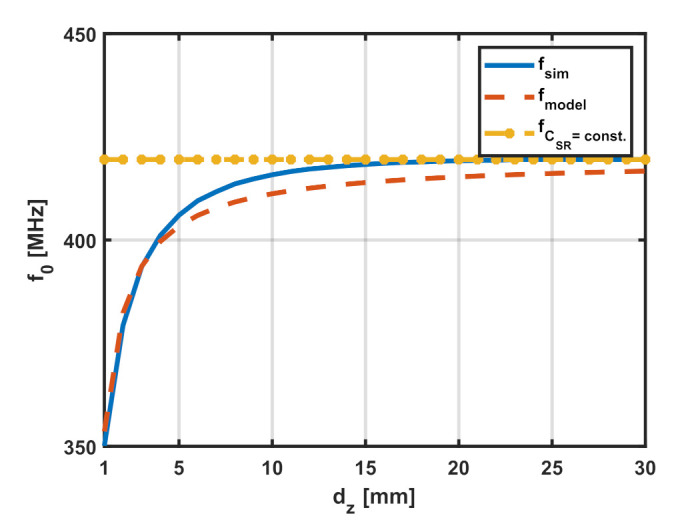
Comparison between the resonant frequency of the SR obtained from numerical simulations and those from the corrected equivalent model. The resonant frequency from the original model is shown by the yellow line for reference. Results are plotted for $L_{p}=L_{tag}=$ 30 mm and w=s= 2 mm, while varying $d_{z}$.

The resonant frequency can be calculated from the model by substituting the result obtained from (13) in (10), and then evaluating the resonant frequency using (1). The accuracy of the fit can be evaluated by comparing the model resonant frequency with the resonant frequency obtained from the EM simulations at different values of $d_{z}$. The results obtained from the model well match those obtained from the EM simulations, as shown in Figure 12 for the case where $L_{tag}=L_{p}=$ 30 mm, showing a mean error less than 1% over the range of distances analyzed. The percentage error is calculated as per (14) and the results are summarized in Table 2.

(14) $$e=\frac{\left|{f_{0}}_{sim}-{f_{0}}_{model}\right|}{{f_{0}}_{sim}}\times 100.$$

**Figure 13:**
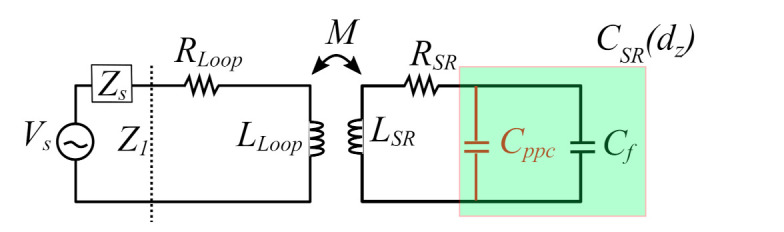
Proposed improved equivalent circuit model, taking into account the additional capacitive coupling ($C_{ppc}$).

It can be deduced from Figure 12 and Table 2 that the model well matches the results from the simulations. The resonant frequency obtained from the original model, which assumes that the equivalent capacitance of the SR is constant, is plotted (yellow line) in the same figure showing the significant error at small values of $d_{z}$ compared to the results from the proposed model, whereas the maximum error in the estimation of the resonant frequency using the original model is equal to 19.9%, compared to 1.1% using the corrected model. The proposed model, shown in Figure 13 by its equivalent circuit, provides a method to predict the decrease in the resonant frequency as the tag moves closer to the loop due to the increased capacitance introduced by the parallel plate capacitor effect which depends on the value of $d_{z}$.

## 5. Experimental Validation

This section is dedicated to experimentally verifying the results previously obtained using simulations and validating the conclusions inferred from the novel fitting model for the additional capacitance presented in the previous section. For this purpose, an experimental prototype of the sensor was realized on an FR-4 substrate ($\epsilon_{r}=4.3$tanδ=0.03) with the following parameters: $L_{p}=22$ mm, $L_{tag}=18$ mm, w= 1.5 mm, s=2 mm, and N=2 turns. It is worth remembering at this point that the shift in the resonant frequency of the tag at small distances is maximized for $L_{p}\approx L_{tag}$. Therefore, the loop antenna and the SR tag are designed such that the $d_{z}$-dependent shift in resonant frequency of the tag at small values of $d_{z}$ is significant so that the proposed additional capacitance model can be verified.

**Figure 14:**
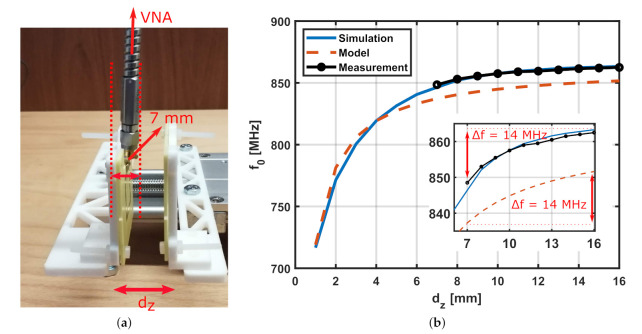
(**a**) Experimental setup showing the minimum value of $d_{z}$ that can be measured due to the size of the soldered SMA connector. (**b**) Comparison between the resonant frequency of the SR obtained from numerical simulations, equivalent circuit model, and experimentally. Results are plotted for a sensor prototype with $L_{p}=22$ mm, $L_{tag}=18$ mm, w=1.5 mm, *s* = 2 mm, and N=2 turns while varying $d_{z}$.

Firstly, an EM simulation was carried out to numerically obtain the resonant frequencies (frequency of maximum real input impedance) and the amplitude at resonance, similar to what was performed in Section 4. Then, the fitting procedure described in Figure 10 was followed to retrieve the effective equivalent area for the sensor with these parameters so that the additional distance-dependent capacitive coupling can be accounted for, as described in (10). The experimental setup for measurements was designed such that the loop antenna is connected through a soldered SMA connector to the Anritsu Shockline MS46524B vector network analyzer (VNA) for signal generation and measurement. The experimental setup for measurement is shown in Figure 14a, where the normal distance $d_{z}$ between the loop and the SR was varied from 7 mm to 16 mm with a step of 1 mm. The minimum value of $d_{z}$ that can be measured using this setup without risk of contact between the tag and the SMA connector is 7 mm, as shown by the dashed lines in Figure 14a.

The results are summarized in Figure 14b, where the resonant frequency of the SR tag ($f_{0}$) is plotted against the $d_{z}$. The resonant frequency obtained from the proposed additional capacitance model (dashed orange line) is compared to those from numerical simulations (solid blue line). It can be observed from the plot that the model well matches the simulation results for a sensor with different parameters than the ones in Figure 12. Furthermore, the good match between the experimentally obtained resonant frequency (black solid line) and the one retrieved from numerical simulations verifies the accuracy and validity of the developed model for EM simulation. Finally, the zoomed-in portion of Figure 14b shows more clearly how the measured resonant frequency decreases at small values of $d_{z}$ and how the fitted model was used to accurately predict the decrease in the resonant frequency of the SR (Δf = 14 MHz) as $d_{z}$ decreases from 16 to 7 mm.

## 6. Conclusions

A tutorial for designing distance sensors based on spiral resonators (SRs) is provided in this paper. The proposed sensor is composed of an SR tag that is interrogated by a microstrip probing loop that is the reader’s antenna. The distance is obtained simply by carrying out a one-port measurement of the real part of the input impedance at the terminals of the probing loop. The real input impedance is inversely proportional to the distance between the SR tag and the probe. This article provides a thorough analysis of the effect of the design parameters of the sensors on the performance, namely, the sensing range, sensitivity, and operating frequency. The results reported herein can be used by designers to quickly reach a preliminary design according to the specific application constraints and requirements. The design process starts by defining the required operating frequency, range, and sensitivity. The designer can optimize the SR geometrical parameters to obtain SR resonance at the desired frequency; this is followed by the design of the probing loop. Two factors must be considered when designing the probe; firstly, its self-resonant frequency must be significantly higher than that of the SR (the desired operating frequency), and, secondly, the size of the probing loop influences the range and sensitivity for distance measurement using the single-frequency interrogation technique described in this work. Finally, the phenomenon of resonant frequency shift occurring when the normal distance between the SR and the probing loop is small is investigated. It has been shown that the equivalent circuit models in the literature for SR sensors fail to take this frequency shift into account. Therefore, a corrected circuit model, based on modeling the capacitive coupling between the SR and the probing loop at small distance as a parallel plate capacitor, has been proposed. The results from this model well match the results obtained from EM simulations and experimental measurements. This model is particularly useful for applications requiring displacement measurement at small distances.

## Figures and Tables

**Table 1:** Fitted equivalent conductor strip area for the additional capacitance for the sensor with $L_{tag}=$ 30 mm.

Parameter	$L_{p}=$ 15 mm	$L_{p}=$ 20 mm	$L_{p}=$ 25 mm	$L_{p}=$ 30 mm	$L_{p}=$ 35 mm	$L_{p}=$ 40 mm	$L_{p}=$ 50 mm
$\tilde{A}\;\left[m^{2}\right]$	$9\times 10^{-6}$	$16.8\times 10^{-6}$	$30.3\times 10^{-6}$	$39.6\times 10^{-6}$	$33.2\times 10^{-6}$	$26.7\times 10^{-6}$	$21.7\times 10^{-6}$

**Table 2:** Mean and maximum percentage error for the resonant frequency estimation using the corrected circuit model for $L_{tag}=$ 30 mm and different $L_{p}$ sizes.

$L_{p}$	15 mm	20 mm	25 mm	30 mm	35 mm	40 mm	50 mm
$e_{mean}$	0.2%	0.4%	0.7%	0.9%	0.6%	0.5%	0.5%
$e_{max}$	0.5%	0.7%	1.1%	1.1%	1.2%	1.3%	1.4%

## References

[1] Chen T., Li S., Sun H. (2012). Metamaterials Application in Sensing. Sensors.

[2] Schueler M., Mandel C., Puentes M., Jakoby R. (2012). Metamaterial inspired microwave sensors. IEEE Microw. Mag..

[3] Costa F., Genovesi S., Borgese M., Michel A., Dicandia F.A., Manara G. (2021). A Review of RFID Sensors, the New Frontier of Internet of Things. Sensors.

[4] Martín F., Vélez P., Gil M. (2020). Microwave Sensors Based on Resonant Elements. Sensors.

[5] Pendry J., Holden A., Robbins D., Stewart W. (1999). Magnetism from conductors and enhanced nonlinear phenomena. IEEE Trans. Microw. Theory Tech..

[6] Ossa-Molina O., Duque-Giraldo J., Reyes-Vera E. (2021). Strain Sensor Based on Rectangular Microstrip Antenna: Numerical Methodologies and Experimental Validation. IEEE Sens. J..

[7] Paredes F., Herrojo C., Martín F. (2021). 3D-Printed Quasi-Absolute Electromagnetic Encoders for Chipless-RFID and Motion Control Applications. Electronics.

[8] Albishi A.M., Alshebeili S.A., Ramahi O.M. (2021). Three-Dimensional Split-Ring Resonators-Based Sensors for Fluid Detection. IEEE Sens. J..

[9] Mata-Contreras J., Herrojo C., Martín F. (2017). Application of Split Ring Resonator (SRR) Loaded Transmission Lines to the Design of Angular Displacement and Velocity Sensors for Space Applications. IEEE Trans. Microw. Theory Tech..

[10] Genovesi S., Costa F., Borgese M., Dicandia F.A., Manara G. (2018). Chipless Radio Frequency Identification (RFID) Sensor for Angular Rotation Monitoring. Technologies.

[11] Dalgac S., Akdogan V., Kiris S., Incesu A., Akgol O., Unal E., Basar M.T., Karaaslan M. (2021). Investigation of methanol contaminated local spirit using metamaterial based transmission line sensor. Measurement.

[12] Al-Duhni G., Wongkasem N. (2021). Metal Discovery by Highly Sensitive Microwave Multi-Band Metamaterial-Inspired Sensors. Prog. Electromagn. Res. B.

[13] Carr A.R., Patel Y.H., Neff C.R., Charkhabi S., Kallmyer N.E., Angus H.F., Reuel N.F. (2020). Sweat monitoring beneath garments using passive, wireless resonant sensors interfaced with laser-ablated microfluidics. NPJ Digit. Med..

[14] Liu Y., Wang M., Yu M., Xia B., Ye T.T. Embroidered Inductive Strain Sensor for Wearable Applications. Proceedings of the 2020 IEEE International Conference on Pervasive Computing and Communications Workshops (PerCom Workshops).

[15] Mahmood M.F., Mohammed S.L., Gharghan S.K., Zubaidi S.L. Wireless Power Transfer based on Spiral–Spider Coils for a Wireless Heart Rate Sensor. Proceedings of the 2020 13th International Conference on Developments in eSystems Engineering (DeSE).

[16] Wang H., Li W., Feng Z. (2015). A Compact and High-Performance Eddy-Current Sensor Based on Meander-Spiral Coil. IEEE Trans. Magn..

[17] Elgeziry M., Costa F., Genovesi S. Distance sensing using spiral resonators. Proceedings of the 2021 XXXIVth General Assembly and Scientific Symposium of the International Union of Radio Science (URSI GASS).

[18] Djuric S.M. (2014). Performance Analysis of a Planar Displacement Sensor With Inductive Spiral Coils. IEEE Trans. Magn..

[19] Su S., Zhang L., Guo Y., Lu F., Tan Q., Xiong J. (2018). Displacement Measurement Realized by Near-field Coupling between Multiple Coils. Sens. Mater..

[20] Kumar P., Ali T., Pai M.M.M. (2021). Electromagnetic Metamaterials: A New Paradigm of Antenna Design. IEEE Access.

[21] Bilotti F., Toscano A., Vegni L. (2007). Design of Spiral and Multiple Split-Ring Resonators for the Realization of Miniaturized Metamaterial Samples. IEEE Trans. Antennas Propag..

[22] Brizi D., Fontana N., Costa F., Monorchio A. (2019). Accurate Extraction of Equivalent Circuit Parameters of Spiral Resonators for the Design of Metamaterials. IEEE Trans. Microw. Theory Tech..

[23] Jow U.M., Ghovanloo M. (2007). Design and Optimization of Printed Spiral Coils for Efficient Transcutaneous Inductive Power Transmission. IEEE Trans. Biomed. Circuits Syst..

[24] Baena J., Bonache J., Martin F., Sillero R., Falcone F., Lopetegi T., Laso M., Garcia-Garcia J., Gil I., Portillo M. (2005). Equivalent-circuit models for split-ring resonators and complementary split-ring resonators coupled to planar transmission lines. IEEE Trans. Microw. Theory Tech..

[25] Mohan S., del Mar Hershenson M., Boyd S., Lee T. (1999). Simple accurate expressions for planar spiral inductances. IEEE J. Solid-State Circuits.

[26] Cheng Y., Shu Y. (2014). A New Analytical Calculation of the Mutual Inductance of the Coaxial Spiral Rectangular Coils. IEEE Trans. Magn..

[27] Elgeziry M., Costa F., Genovesi S. (2021). Wireless Monitoring of Displacement Using Spiral Resonators. IEEE Sens. J..

[28] Schormans M., Valente V., Demosthenous A. (2018). Practical Inductive Link Design for Biomedical Wireless Power Transfer: A Tutorial. IEEE Trans. Biomed. Circuits Syst..

[29] Ko W.H., Liang S.P., Fung C.D.F. (1977). Design of radio-frequency powered coils for implant instruments. Med Biol. Eng. Comput..

[30] Moreton G., Meydan T., Williams P. (2018). Using finite element modelling and experimental methods to investigate planar coil sensor topologies for inductive measurement of displacement. AIP Adv..

[31] Horestani A.K., Fumeaux C., Al-Sarawi S.F., Abbott D. (2013). Displacement Sensor Based on Diamond-Shaped Tapered Split Ring Resonator. IEEE Sens. J..

[32] Yuan C., Trick T. (1982). A simple formula for the estimation of the capacitance of two-dimensional interconnects in VLSI circuits. IEEE Electron Device Lett..

[33] Majumdar A., Cunningham J.E., Krishnamoorthy A.V. (2010). Alignment and Performance Considerations for Capacitive, Inductive, and Optical Proximity Communication. IEEE Trans. Adv. Packag..

